# Generalized and Specific Problematic Internet Use in Central Siberia Adolescents: A School-Based Study of Prevalence, Age–Sex Depending Content Structure, and Comorbidity with Psychosocial Problems

**DOI:** 10.3390/ijerph19137593

**Published:** 2022-06-21

**Authors:** Sergey Tereshchenko, Edward Kasparov, Nadezhda Semenova, Margarita Shubina, Nina Gorbacheva, Ivan Novitckii, Olga Moskalenko, Ludmila Lapteva

**Affiliations:** Federal Research Center “Krasnoyarsk Science Center of the Siberian Branch of the Russian Academy of Sciences”, Research Institute of Medical Problems of the North, 660022 Krasnoyarsk, Russia; kasparov_impn_1@mail.ru (E.K.); snb237@gmail.com (N.S.); marg-shubina@mail.ru (M.S.); n-n-gorbacheva@yandex.ru (N.G.); ivan_nov_@mail.ru (I.N.); olga_olgaol@mail.ru (O.M.); yara3011@yandex.ru (L.L.)

**Keywords:** adolescents, internet addiction, problematic internet use, game addiction, social media addiction, Russia

## Abstract

We aimed to assess the prevalence, content structure and, psychological comorbidity of PIU in Russian adolescents. In addition, the design of our research provided an opportunity to compare demographic and psychological patterns of different forms of PIU: generalized (PIUgen) and specific problematic video game use (PUgame), as well as problematic social media use (PUsocial). Methods: This is a one-stage cross-sectional observational study of school sampling in three major Siberian cities. A total of 4514 schoolchildren aged 12–18 (mean age 14.52 ± 1.52 years) were surveyed. The Chen Internet Addiction Scale, the Game Addiction Scale for Adolescents, and the Social Media Disorder Scale were used to identify PIU and its types. Results: The prevalence of PIUgen among adolescents in Central Siberia was 7.2%; the prevalence of PUgame was 10.4%; the prevalence of PUsocial was 8.0%. The results of structural equation modelling, as well as the correlation analysis data, suggest two possible patterns of psychosocial problems with PIU—the first one is characteristic of both PIUgen and PUsocial. The second one—which is significantly different—is characteristic of PUgame. Conclusions: Urban adolescents in Central Siberia do not differ significantly from their Asian and European peers. Our findings support the concept of rejecting the term “generalized PIU” as a single psychological construct.

## 1. Introduction

The last two decades have been characterized by the avalanche-like increase in Internet use in all social groups, especially among adolescents and young adults [1]. The phenomenon of Internet addiction, or “pathological/compulsive Internet use,” occurs in a number of predominantly adolescent and young adult Internet users; it is characterized by loss of control over online activities and a compulsive craving for various internet activities and is often a cause of a wide range of psychosocial and psychosomatic problems.

First described in the mid-1990s, the phenomenon of Internet addiction [2,3,4] has generated numerous scientific, clinical, and social debates from its emergence to the present. From the point of view of classical psychology and psychiatry, addictive online behavior is a relatively new phenomenon that has, to date, no generally accepted formal definition. Some interchangeable terms can be found in the specialized literature, such as the recently proposed “problematic interactive media use” [5], as well as the more traditional “problematic Internet use” (PIU), “pathological Internet use”, “compulsive Internet use”, and finally “Internet addiction.” All of these are “umbrella” terms reflecting generalized problematic Internet use (PIUgen), without reference to specific content. At the present time, five specific types of online activity can be considered potentially addictive: problematic video game use (PUgame), problematic social media use (PUsocial), problematic Internet pornography use, Internet gambling, and compulsive Internet searching and surfing [6]. Only one of these five addictive types of behavior, namely PUgame, is now officially considered to be a mental disorder (Internet Gaming Disorder, DSM-5, American Psychiatric Association, 2013; Gaming Disorder, ICD-11, 2019; available at https://psychiatry.org/psychiatrists/practice/dsm (accessed on 19 June 2020) and https://icd.who.int/en (accessed on 19 June 2020), respectively). 

The PIU prevalence figures with respect to adolescents, as published in the global literature, vary widely, from 1% to 50%, depending on the explored ethno-social groups, the diagnostic criteria, and the questionnaires [1,7]. In Europe, for instance, the prevalence of PIU among adolescents is 1–11%, with an average of 4.4% [8]. In the United States, the prevalence of PIU in the total sample is 0.3–8.1% [9]. At the same time, the prevalence of PIU in Asian countries (China, South Korea, Singapore, etc.) is much higher among adolescents and young adults, ranging from 8.1 to 26.5% [10,11]. 

A large number of studies have convincingly demonstrated a strong comorbidity of PIU with a wide range of psychopathological conditions [12,13]. For instance, a meta-analysis by Ho et al. [14] demonstrated the comorbidity of PIU with depression (Odds Ratio (OR) = 2.77, Confidence Interval (CI) = 2.04–3.75); anxiety disorders (OR = 2.70, CI = 1.46–4.97); and attention deficit hyperactivity disorder (OR = 2.85, CI = 2.15–3.77). Carli et al., in their systematic review, showed that depressive disorder and attention deficit hyperactivity disorder have the highest association with PIU, while anxiety disorder, obsessive-compulsive disorder, social phobia, and aggressive behavior have a smaller but significant association. The same conclusions were reached in a recent systematic review [12]. The research of Durkee et al. [15], which included a representative sample of 11,356 adolescents from 11 European countries, conclusively showed a correlation between PIU and self-harming and suicidal behavior, as well as depression and anxiety. The same findings were obtained in a recent study by Jiang et al. [16]. At the same time, it must be emphasized that the true causal links between PIU and psychosocial problems remain unknown. Both the prior influence of psycho-emotional problems on the emergence of addiction and the reverse—the emergence of problems due to staying online excessively—are possible.

In addition, PIU in adolescents was found to be associated with chronic conditions (OR = 1.58, CI = 1.11–2.23); back pain (OR = 1.46, CI = 1.04–2.05); overweight (OR = 1.74, CI = 1.03–2.93); musculoskeletal pain (OR = 1.36, CI = 1.00:1.84); and sleep disturbance (OR = 2.16, CI = 1.62–2.88) [17,18]. 

As far as we know, Russia is a “blank spot” with regard to the scientific findings described above—so far, no data on the prevalence, structure, and psychiatric comorbidity of PIU in Russian adolescents have been published in the global English-language literature. This is especially interesting for Siberia, a multi-ethnic Asian region with a cold climate and short daylight hours in winter, which can potentially affect the frequency and duration of Internet usage. We believe that such data should be available to the global scientific community, at least in the context of comparing different populations.

An important aspect and trend in contemporary PIU research is the attempt to move away from the study of solely generalized, undifferentiated PIU content, towards the analysis of specific types of PIU, such as PUgame and PUsocial [19,20]. It remains unclear to what extent these types of pathological Internet activity are related to PIUgen and to each other. Sample analysis, using simultaneous screening tools for PIUgen, PUgame, and PUsocial, could represent a new approach in this direction [21]. We are aware of only two studies with a simultaneous analysis of PIUgen, PUgame, and PUsocial; both of them were based on adult population groups [22,23]. Adolescent population groups have not been investigated in studies with a similar design, which necessitates the need for such analysis.

The present study aims to be the first attempt in the global English-language literature to assess the prevalence, content structure, and psychological comorbidity of PIU among Russian adolescents by the example of unbiased school sampling. Furthermore, the design of this study provides an opportunity to systematically explore how different forms of PIU (PIUgen, PUgame, and PUsocial) relate to each other and to what extent they are similar or different in prevalence, demographic characteristics, and psychological patterns.

## 2. Materials and Methods

### 2.1. Participants

The research represents a cross-sectional observational study of an unbiased school sample in three large cities of Central Siberia. The research objects were represented by adolescents aged 12–18 (*n* = 4514; mean age 14.52 ± 1.52 years, boy/girl ratio 46.4%/53.6%). They were the students of 10 comprehensive schools in Krasnoyarsk, Russia (*n* = 2901), 4 comprehensive schools in Abakan, Russia (*n* = 1400) and 2 comprehensive schools in Kyzyl, Russia (*n* = 213).

### 2.2. Measurement

After obtaining informed parental consent, the learners were notified that the survey was voluntary and confidential, and they were asked to complete self-report questionnaires in paper form in a common classroom area for 45 min. The survey was conducted in spring 2019. The study was approved by the Ethics Committee of the Federal Research Centre “Krasnoyarsk Science Centre of the Siberian Branch of the Russian Academy of Sciences”.

#### 2.2.1. Internet Addiction Measurement

To identify the presence of Internet-dependent behavior, the Chen Internet Addiction Scale (CIAS) was used [24]. The Russian version of the CIAS was adapted by Malygin et al. and can be freely downloaded from http://www.medpsy.ru/library/library135.pdf (accessed on 19 June 2020). The CIAS covers five symptomatic criteria of addictive behavior; among them are compulsive symptoms, withdrawal symptoms, signs of tolerance, the presence of psychological or somatic problems, and time management difficulties. The questionnaire comprises 26 statements, each rated according to the 4-score scale: “not at all suitable” (1 point), “slightly suitable” (2 points), “partially suitable” (3 points), and “totally suitable” (4 points). The overall CIAS ≥ 65 was treated as the presence of PIUgen.

The content consumption patterns in adolescents with Internet addiction were analyzed using the Russian-language version of the “Game Addiction Scale for Adolescents” (GASA) questionnaire [25] and the “Social Media Disorder Scale” (SMDS) questionnaire [26]. The Russian versions of the GASA and SMDS questionnaires were prepared and validated by the authors of this publication. 

The GASA questionnaire comprises 7 questions relating to behavioral disorders in adolescents caused by an excessive preoccupation with Internet gaming. Each question was rated on a 5-score scale: “never” (0 points), “rarely” (1 point), “sometimes” (2 points), “often” (3 points), “very often” (4 points). According to the criteria proposed by the authors [25], two options were identified—a polythetic format of addiction or PUgame (if any 4 or more of the 7 questions were answered as “sometimes”, “often”, or “very often”) and a monothetic format of addiction (if all the questions were answered “sometimes” or “often”, or “very often”). 

The SMDS questionnaire consists of 9 questions relating to behavioral disorders caused by an excessive use of social networking sites. Each question has two answer options: “no” and “yes”. Five or more positive answers out of nine points to the presence of PUsocial [26]. A recent cross-national analysis of adolescents’ psychometric characteristics in 44 countries showed a high level of validity and reliability for the SMDS [27].

#### 2.2.2. Psychosocial Problems Assessment

The Strengths and Difficulties Questionnaire (SDQ) was developed by Goodman et al. [28] as a brief psychopathological screening tool that has been recommended for the detection and classification of psychosocial problems in adolescents. The SDQ is widely used today, both in clinical practice and in scientific research, and is characterized by concision coupled with reliability and a possibility to assess various aspects of an adolescent’s psychosocial condition. An indisputable advantage of the SDQ is its wide accessibility—it has been translated into more than 80 languages, and it is freely available on the developers’ website (https://sdqinfo.org, accessed on 19 June 2020), which allows for cross-cultural comparison. The Russian-language version of the SDQ has been thoroughly validated by Ruchkin et al. [29] and Slobodskaya et al. [30] through a sample of Siberian schoolchildren (Novosibirsk, Russia). 

A one-sided, self-rated SDQ for children aged 12–17 was used. The SDQ consists of 25 statements relating to the adolescent’s mischief and socially acceptable behavior over the previous 6 months. The answers are scored on a 3-point scale (0 = not true, 1 = somewhat true, and 2 = certainly true; the points are assigned in forward or reverse order for each question in accordance with the authors’ instructions [28]) and are grouped under five scales: Emotional symptoms, Conduct problems, Hyperactivity/inattention, Peer problems, and Prosocial behavior. As provided by the instructions of the questionnaire authors [28], the statement points are summed up and grouped to calculate an index for every scale: Emotional symptoms—statements 3, 8, 13, 16, and 24; Conduct problems—5, 7, 12, 18, and 22; Hyperactivity/inattention—2, 10, 15, 21, and 25; Peer problems—6, 11, 14, 19, and 23. The score summary value reflects the severity of the problems in a particular area for a particular adolescent. In addition, a resulting scale, the Total Difficulties Score, can be calculated by summing up the first four scaled scores. Separately, the Prosocial Behavior Score is calculated based on the summary score of statements 1, 4, 9, 17, and 20. We used the Russian version of the questionnaire, which can be freely downloaded from the developers’ website https://sdqinfo.org/ (accessed on 19 June 2020).

The psychometric characteristics of the questionnaires included in the study are presented in Table 1.

### 2.3. Statistical Analysis

The statistical analysis of the results was performed using the IBM SPSS Statistics for Windows, v20.0, with the Statistical Package for the Social Sciences and AMOS 21.0 (IBM Corp., Armonk, NY, USA). The confirmatory factor analysis was carried out in the statistical computing system R [31] and the Lavaan package [32], implemented in the Jamovi graphical interface (ver. 2.0 [33]). The quality of the models’ fit with the empirical data was assessed using the following indicators: χ^2^, root mean square error of approximation (RMSEA), the comparative fit index (CFI), and the Tucker–Lewis index (TLI). The association of different types of problematic Internet use with the relevant variables was assessed with the use of multiple regression analysis within structural equation modelling. The regression coefficients were estimated while controlling all the other predictors in the model. The odds ratios (ORs) were calculated by multiple logistic regression, with adjustment for covariates. The strength of the relationship between the two variables was determined using Spearman’s rank correlation coefficient (*r*). The Student’s *t*-test was used to compare the quantitative data. The comparison of groups by the qualitative binary data was performed using the Pearson χ^2^ test with the Yates correction.

## 3. Results

### 3.1. Descriptive Statistics

The original sample comprised 4838 students. After removing those who were not able to complete all the sections of the questionnaire (*n* = 324, 6.7%), the sample comprised 4514 adolescents. Thus, the response rate in this study amounted to 93.3%.

The demographic characteristics of the sampled adolescents in the present study, along with the main descriptive statistics of the generalized and specific PIU parameters (PIUgen, PUgame, and PUsocial) used in the research, as well as the mean SDQ scales values, are presented in Table 2. The mean age of the 4514 tested adolescents was 14.52 ± 1.52 years, with a boy/girl ratio of 2092 (46.4%)/2422 (53.6%).

### 3.2. Prevalence and Age–Sex Dependent Content Structure

The prevalence of PIUgen, as estimated based on the CIAS questionnaire, was 7.2% of the total sample and was higher in the girls than in the boys (8.9% vs. 5.2%, *p* < 0.001). The prevalence of PUgame, as estimated based on the GASA questionnaire results, was 10.4% of the total sample, being more than twofold higher in the boys compared to the girls (15.7% vs. 5.9%, *p* < 0.001). The prevalence of PUsocial, as estimated based on the SMDS questionnaire, was 8.0% of the total sample, being more than three times higher in the girls compared to the boys (11.8% vs. 3.5%, *p* < 0.001). Compared with the boys, the girls in our sample scored higher on the emotional symptoms and prosocial behavior scales of the SDQ (Table 2). Similar gender differences were described in other adolescent populations surveyed with the SDQ [29,34]. 

The age-specific characteristics of generalized and specific PIU (PIUgen, PUgame, and PUsocial), with stratification by gender, are presented in Table 3. The boys showed no age differences in PIUgen prevalence (5.7% in 12–14-year-olds vs. 4.8% in 15–18-year-olds, *p* = 0.404), whereas the prevalence of PIUgen in the girls increased with age (7.7% in 12–14-year-olds vs. 10.1% in 15–18-year-olds, *p* = 0.042).

The adolescents who had played computer games within the month before the research were more numerous among the boys than the girls (91.6% vs. 65.4%, *p* < 0.001). With age, the number of active computer game users decreased slightly among the boys (92.7% in 12–14-year-olds vs. 89.5% in 15–18-year-olds, *p* = 0.013) and to a greater extent among the girls (71.3% in 12–14-year-olds vs. 59.6% in 15–18-year-olds, *p* < 0.001). Consistently, the PUgame prevalence hardly decreased with age in the boys (17.4% in 12–14-year-olds vs. 14.2% in 15–18-year-olds, *p* = 0.058) and was 1.5 times lower in the older girls than in the younger ones (7.2% in 12–14-year-olds vs. 4.6% in 15–18-year-olds, *p* = 0.008). Interestingly, the relative number of adolescents with marked computer game addiction (7/7 GASA items endorsed) was almost identical in the boys and girls of the younger age group (1.1% vs. 0.8%, *p* = 0.660) and 4 times higher in the boys of the older age group compared to the girls (1.9% vs. 0.5%, *p* = 0.003).

Siberian adolescents, regardless of gender and age, are very active in using the social resource of the Internet: 93.0–97.6% reported using social media over the year. The prevalence of PUsocial decreased with age in both the boys (4.4% in 12–14-year-olds vs. 2.7% in 15–18-year-olds, *p* = 0.043) and the girls (14.0% in 12–14-year-olds vs. 9.6% in 15–18-year-olds, *p* = 0.001). The relative number of adolescents with a strong social media dependence (9/9 SMDS items endorsed) was registered in the interval of 0.3–0.5% and was not dependent on gender or age (Table 3).

**Figure 1:**
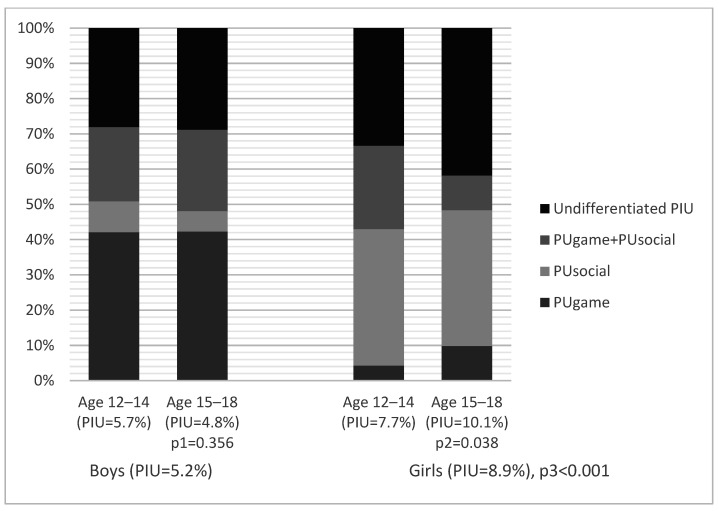
Content consumption patterns among adolescents with positive CIAS test (≥65) by sex and age. Note: p1—Age 12–14 vs. Age 15–18 in boys; p2—Age 12–14 vs. Age 15–18 in girls; p3—boys vs. girls.

The content consumption structure among the adolescents with a positive CIAS test (≥65), as a function of gender and age, is presented in Figure 1. It can be seen from the presented data that at least 60–70% of adolescents with PIUgen, according to the CIAS results, had PUgame, PUsocial, or their combination. Whereas the content consumption structure remained virtually unchanged with age in the boys, the girls showed a significant increase in the relative share of undifferentiated PIU with age (non-PUgame and non-PUsocial among the CIAS positive—2.6% in 12–14-year-old girls vs. 4.2% in 15–18-year-old girls, *p* = 0.003). 

The overlaps between PIUgen, PUgame, and PUsocial are shown in Table 4, Table 5 and Table 6. Only a small proportion of the adolescents demonstrated a combination of PIUgen traits with positive tests for PUgame (2.7%) and PUsocial (3.3%), as well as a combination of PUgame and PUsocial traits (2.4%). However, while the PIUgen+PUgame share accounted for only 26% of the entire group with PUgame, the PIUgen+PUsocial proportion accounted for as much as 41% of the entire group with PUsocial. Thus, PUsocial correlates more with the positive results, confirming the presence of PIUgen as assessed by the CIAS questionnaire.

### 3.3. Comorbidity Problematic Internet Use with Psychosocial Problems

#### 3.3.1. Logistic Regression Analysis

The results of the logistic regression analysis showed a high degree of PIU associated with adolescent psychosocial problems (Table 7). The highest association for all the PIU detection questionnaires was found with the presence of emotional problems and hyperactivity. The highest degree of association was found for the CIAS questionnaire assessing PIUgen: OR = 3.65 (CI 2.89–4.64) for emotional symptoms and OR = 4.56 (CI 2.89–4.64) for hyperactivity. The same predictors were highly associated with the presence of PUsocial and—to a somewhat lesser extent—with the presence of PUgame (Table 7). The only factor that did not show any association with PIU was the low level of prosocial behavior in the presence of PUsocial.

#### 3.3.2. Correlation Analysis

The correlation analysis results of the explored psychometric characteristics are presented in Table 8. As with the results of the logistic regression, the correlation analysis showed a high association of emotional symptoms and hyperactivity scales with the presence of PIUgen and PUsocial. At the same time, the presence of PUgame demonstrated a significantly lower Spearman’s rank correlation coefficient with the CIAS scales, which in no case exceeded the minimal level of association (>0.3). We also found no significant correlation between the total GASA scores and the CIAS scales (r = 0.292, *p* < 0.001) and the SMDS (r = 0.159, *p* < 0.001).

#### 3.3.3. Multiple Regression Analysis within Structural Equation Modelling

**Figure 2:**
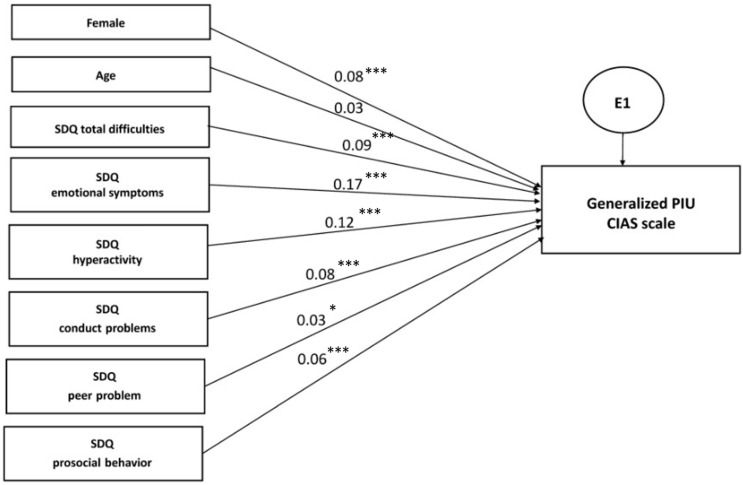
Multiple regression model for generalized problematic Internet use (PIUgen), *n* = 4514. Note: standardized regression weights are shown. Variables were coded as: boys—1, girls—2; age 2–14—1, age 15–18—2; model fit parameters: CFI = 0.993, TLI = 0.962, RMSEA = 0.036, *p* < 0.001. *: *p* < 0.05; **: *p* < 0.01; ***: *p* < 0.001.

**Figure 3:**
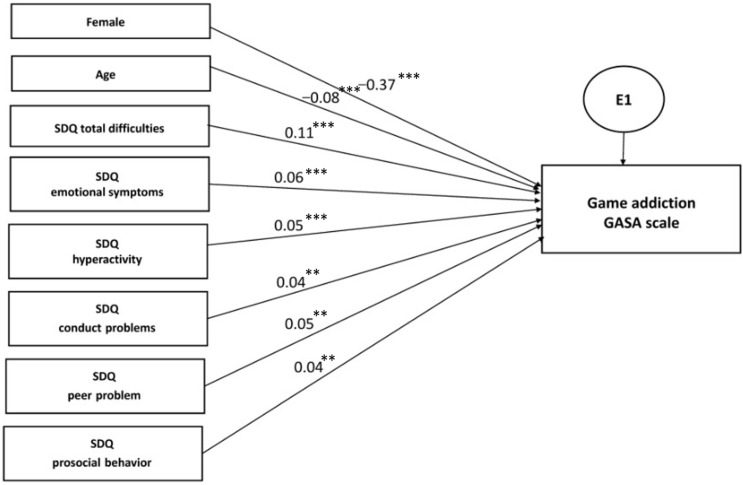
Multiple regression model for problematic video game use (PUgame), *n* = 4514. Note: standardized regression weights are shown. Variables were coded as: boys—1, girls—2; age 2–14—1, age 15–18—2; model fit parameters: CFI = 0.993, TLI = 0.964, RMSEA = 0.036, *p* < 0.001. *: *p* < 0.05; **: *p* < 0.01; ***: *p* < 0.001.

**Figure 4:**
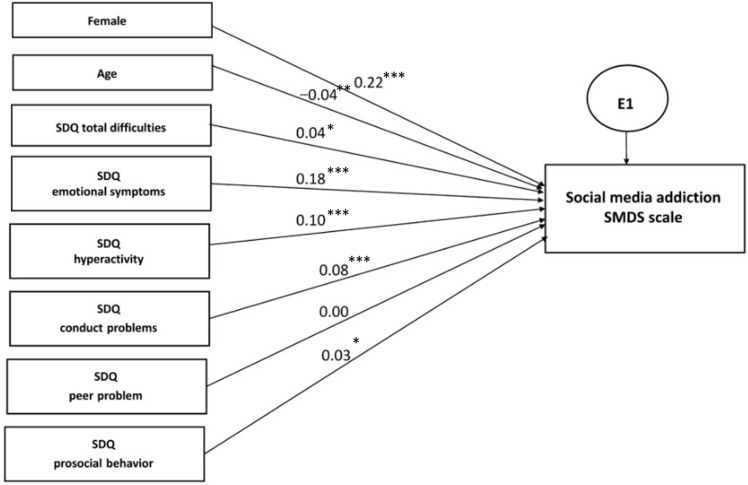
Multiple regression model for problematic social media use (PUsocial), *n* = 4514. Note: standardized regression weights are shown. Variables were coded as: boys—1, girls—2; age 2–14—1, age 15–18—2; model fit parameters: CFI = 0.993, TLI = 0.963, RMSEA = 0.036, *p* < 0.001. *: *p* < 0.05; **: *p* < 0.01; ***: *p* < 0.001.

In order to compare the link of PIUgen, PUgame, and PUsocial with the relevant predictor variables, including the demographic and psychosocial characteristics based on the SDQ, we undertook a separate multiple regression analysis within structural equation modelling (Figure 2, Figure 3 and Figure 4; Table 9, Table 10 and Table 11). The results demonstrated evident and distinct associations of certain predictor variables with the explored outcome variables. Thus, the male gender representatives showed a very high association with PUgame (β = −0.37, *p* < 0.001), whereas the female gender representatives demonstrated associations with PUsocial, although to a lesser extent (β = 0.22, *p* < 0.001). The age, city of residence, and nationality of the adolescents showed a very weak association with all the addiction scales.

Among the SDQ questionnaire scales, the greatest standardized effect was shown by the scales depicting the presence of emotional symptoms and hyperactivity for all the outcome variables. Interestingly, the profile of psychosocial problems was extremely close in PIUgen and PUsocial, while PUgame was not. When analyzing the CIAS scale, the β effect strength was equal to 0.17 (*p* < 0.001) for emotional symptoms and 0.12 (*p* < 0.001) for hyperactivity, while, with regard to the SMDS scale, it was 0.18 (*p* < 0.001) and 0.10 (*p* < 0.001), respectively. At the same time, when the GASA scale results were analyzed, the β coefficient was only 0.06 (*p* < 0.001) for emotional symptoms and 0.05 (*p* < 0.001) for hyperactivity; a nearly equivalent association with PUgame was demonstrated by conduct disorders and peer problems of 0.05 (*p* = 0.006) and 0.04 (*p* = 0.001), respectively. Thus, the associative links were largely similar for PIUgen and PUsocial, but quite different for PUgame.

## 4. Discussion

The PIU prevalence figures for adolescents published globally vary from 1% to 50%, depending on the explored ethno-social groups and the used diagnostic criteria and questionnaires, [1,7]. In Europe, for instance, the prevalence of PIU among adolescents is 1–11%, with an average of 4.4% [8]. In contrast, the PIU prevalence in Asia (India, China, Japan, South Korea, Thailand, and Taiwan) is much higher among adolescents and young adults and ranges from 7.9–49.4% [10,11,35,36]. The meta-analysis data on the prevalence of PIU among those under 25 in the Gulf countries (Bahrain, Iraq, Kuwait, Oman, Qatar, Saudi Arabia, and the United Arab Emirates) also show high values of up to 33% [37]. One may conclude, based on recent major systematic reviews and the meta-analysis data, that the global average prevalence of PIU in adolescents and young adults is about 5%, with some exceedance in Asia and North America [1,38,39,40].

Our data show that the overall prevalence of PIUgen among urban adolescents in Central Siberia is 7.2%, differing little from the global average prevalence of PIU (see above) and from the data previously obtained for the East Asian region with the CIAS questionnaire. For instance, a meta-analysis by Shao et al., using CIAS-based data from three studies of Chinese adolescents, calculated an average prevalence of PIUgen of 9.0% [41]. A recent CIAS testing of 8098 Chinese students aged 17–25 also showed a similar figure on the prevalence of PIUgen of 7.7% [42]. 

In our sample, the PIUgen prevalence, as assessed by the CIAS questionnaire, was higher in the girls than in the boys, which is somewhat contrary to the findings of other studies—a meta-analysis by Wenliang et al. showed that males have a higher propensity for Internet addiction than females [43]. However, this proportion is not universal. As shown by the aforementioned study by Shen et al., based on the CIAS questionnaire, the frequency of PIUgen was slightly higher in girls than in boys (8.2% vs. 7.2%, *p* = 0.11) [42]. A higher prevalence of Internet addiction in the Gulf countries was found in women compared with men (48% vs. 24%, *p* = 0.05) [37]. Finally, a recent CIAS-based Russian testing of adolescents in the Urals also found a higher prevalence of PIUgen in girls compared to boys [31].

We hypothesize that the socio-cultural characteristics of the target population, which determine the predominantly consumed Internet content, can play a significant role in the gender distribution of adolescents with generalized PIU. Possibly, the gender differences we have identified are related to the gender specifics of the content consumption in Siberian adolescents: the girls showed a threefold intensity of social media addiction, whereas the boys demonstrated two times more intensity of gaming addiction (Table 2). As shown below, as the CIAS questionnaire, which is designed to identify generalized Internet addiction that is undifferentiated in content, correlates more with social media addiction, the gender distribution of PIUgen prevalence identified in our study has an obvious logical justification.

As shown in a systematic review by Mihara and Higuchi [44], PUgame prevalence ranges from 0.7% to 27.5% and, as with generalized PIU, is highly dependent on the used questionnaires and the evaluative dependence criteria. As with the case of generalized PIU, the prevalence of PUgame shows a higher prevalence in Asian countries compared to other regions [45]. As shown by our sample, 79.8% of adolescents played computer games during the month before the review, 10.4% were categorized as PUgame adherents (4/7 GASA items endorsed) and 1.1% were characterized as having computer game addiction (7/7 GASA items endorsed; Table 3). Our results are close to those obtained by the authors of the GASA questionnaire for a Dutch adolescent sample (9.3–9.4% in terms of the first mode of assessment and 0.55–0.65% for the second evaluative method) [25] and are also very similar to other population-based studies using GASA in adolescent samples from Hong Kong [46], Finland [46], and Norway [47,48]. As in most other studies [44], the prevalence of PUgame in our sample was significantly higher in the boys when compared to the girls (*p* < 0.001, Table 2). 

According to our data, the prevalence of PUsocial among Siberian adolescents was 8.0%, which is not particularly different from the prevalence data obtained by the authors of the SMDS questionnaire for a Dutch teenage cohort (7.3–11.6) [26], as well as other works using the SMDS: a Dutch sample in a longitudinal study (9.9–10.0%) [49] and a representative sample of 3408 Finnish adolescents with a prevalence of 9.4% [50]. However, the SMDS results for the representative sample of German adolescents (*n* = 1001) were much lower, with a prevalence of 2.6% [51]. Interestingly, the same study also reported a significantly lower prevalence of PUgame (3.5%) compared with our data. This essential distinction may be explained by significant differences in the data collection methodology—the German researchers used a telephone survey and some specific methods to ensure population representativity over a wide socio-geographic range, whereas our research was based on a school survey of adolescents having similar social characteristics. Interestingly, other studies providing representativity also identified significantly lower prevalence rates of PUgame and PUsocial compared with convenience sampling [52,53]. A recent systematic review by Cheng et al. [54] showed a high heterogeneity in PUsocial prevalence, within 5–26%: the major modifying factors comprised the addiction classification method (monothetic/polythetic models and cut-offs) and geographic/cultural factors. The highest prevalence rates of PUsocial are registered in collectivist nations within Asian and African countries [54]. The average PUsocial prevalence in 29 European countries was 7.4% [55].

In our sample of Siberian adolescents, the girls were significantly more likely to meet the criteria of PUsocial presence. At the same time, the aforementioned studies of Dutch adolescents did not find any correlation between the prevalence of PUsocial and gender [26,49]. However, the data presented in several studies correlate with our findings: the PUsocial prevalence in girls was also found in German [51], Hungarian [53], Finnish [50], and South Korean [37] adolescents, as well as in Spanish 17–25-year-olds [56]. Interestingly, gender differences in content consumption persist into adulthood: a survey of 23,533 adults in Norway showed PUgame association with males and PUsocial with females [57]. 

As to the age-related dynamics of addiction, our data showed that the prevalence of PUgame decreased weakly with age in the boys (*p* = 0.058) and lowered significantly in the girls (*p* = 0.008). The prevalence of PUsocial decreased with age in both the boys (*p* = 0.043) and the girls (*p* = 0.001). A higher prevalence of PUsocial (but not PUgame) in younger learners, compared to the older ones, was also shown in a recent survey of German schoolchildren [51]. The same, but less pronounced, tendency was demonstrated in a recent study of a representative sample of German adolescents [21]. 

These facts may indicate a low degree of symptom stability and remission in a significant proportion of adolescents. As our study is not longitudinal and representative research, our data on the age-related dynamics of generalized and specific PIU should be interpreted with a certain caution. However, some longitudinal studies undertaken for other populations show similar dynamics—the prevalence of PIU decreases slightly with age, although longitudinal stability for PUgame in adolescence can be quite high, while the remission rate may be low [44,45]. The findings on the natural course and stability of PIU symptoms are very important. This was emphasized by a research team that developed the PUgame diagnostic criteria for DSM-5 [58]. If the core symptoms of generalized and specific PIU are transient and have a self-repairing nature, these conditions should not be treated as a separate clinical area. We deem that the introduction of an additional criterion for Internet addiction in adolescents is worth discussing—the stability of symptoms, e.g., within 3 years, to establish a clinical diagnosis. In the absence of this criterion and the presence of other symptoms, adolescents should be classified as an at-risk group, without a definite clinical addiction diagnosis, in order to avoid psychiatric stigmatization.

In our opinion, the data on PIU structure age dynamics, presented in Figure 1, seem to be quite interesting. As for the boys, we recorded a fairly stable structure not changing within the age interval of 12–18. At the same time, the share of PIU not differentiated in this research (non-PUgame and non-PUsocial PIU) increased in the girls significantly with age. At present, we cannot judge definitely on the older girls’ change of interests—into which area of Internet activity they are shifting. These can represent online chatting, consumption of audio and video content available exclusively on the Internet, online shopping, permanent news searches in fear of missing out, compulsive absent-minded Internet surfing, the use of the Internet for educational purposes, or a combination of the above. This unresolved issue should be a subject of further research. 

The analysis of psychosocial problems in adolescents with PIU showed that the major identified association involved the presence of emotional problems, which may be, to a certain extent, due to the presence of anxiety-depressive disorders, as well as hyperactivity problems, which may indicate that some adolescents have attention deficit hyperactivity disorder. Our findings are consistent with those of numerous other studies, both those involving the SDQ questionnaire [42,59,60] and those based on generalized analysis [45,61]. It is currently unknown whether anxiety-depressive disorders and attention deficit hyperactivity disorder are precedents of PIU or whether the symptoms appear or increase as a result of excessive use of the Internet—or whether we are dealing with a bilateral influence. Independent comorbidity with the influence of certain common risk factors provoking these disorders is not excluded as well. In order to clarify these issues, representative longitudinal research of different populations and age–sex groups, including content consumption, is essential. 

Interestingly, peer problems seemed to be characteristic of PIUgen and PUgame, excluding PUsocial, which may testify to the greater socialization and empathy of adolescents actively engaged in social media activities (Figure 2, Figure 3 and Figure 4; Table 9, Table 10 and Table 11). Similar results were also obtained in a recent study by Sümen and Evgin [60]. 

The results of structural equation modelling, as with our data derived from logistic regression and correlation, suggest two patterns of psychosocial problems in the case of PIU; the first one is characteristic of both PIUgen and PUsocial and the second one, which is significantly different, is characteristic of PUgame.

The relationship between PIUgen and PUgame has been hotly debated in the literature in recent years: most experts believe that these are conceptually distinct psychological constructs [19,20]. Our data, as with the data of other empirical studies, support this view. For instance, Király et al. convincingly showed the conceptual differences between PIUgen and PUgame and supported the need for a differentiated approach to these disorders [62]. The same data were obtained in the course of an analysis of PIUgen and PUgame in medical school students; the authors believe that PUgame cannot be regarded as a subtype of PIUgen [63]. 

Moreover, our data, along with the data of numerous other research works, point not only to conceptual differences between PUgame and PUsocial but also to the fact that the existing questionnaires identifying PIUgen are significantly more correlated with social network addiction rather than computer game addiction. For instance, Montag et al. demonstrated in their analysis of pooled data for young adult populations in Germany, Sweden, Taiwan, and China that it is PUsocial, but not PUgame, that is associated with PIUgen [22]. Lopez-Fernandez, in a simultaneous survey of a sample of Belgian adults, with respect to PIUgen, PUgame, and PUsocial, obtained results completely similar to ours: PIUgen showed a strong association with PUsocial and a weak association with PUgame, with both specific PIU types being independent of each other [23]. Andreassen et al., in their analysis of the association of different types of PIU with psychiatric disorders (attention deficit hyperactivity disorder, obsessive-compulsive disorder, and anxiety disorder) in adults, also showed greater correlation values for PUsocial compared to PUgame, although depressive disorder was more specific to PUgame [57]. As shown by Wartberg et al., clinically significant depression in an adolescent population of German schoolchildren was identified in 34.6% of cases with PUsocial and only in 14.3 of cases with PUgame [51]. However, contrasting empirical evidence exists as well. For instance, a recent analysis of a representative sample of German adolescents showed no difference in the incidence of psychosocial problems between the PUgame and PUsocial groups, which is explained by the high prevalence of recently embedded gaming content in social networks, as viewed by the authors [21]. No significant differences were found in the frequency of anxiety and depression between the PUgame and PUsocial groups and in a study of English teenagers [64]. 

Although there are some inconsistencies in the empirical data, we believe that our findings, as well as the data of other researchers, support the concept of rejecting the term PIU as a single psychological construct. Future research efforts should focus on studying specific forms of adolescents’ addictive online behavior. 

This study has some limitations. The research was not anonymous; the questionnaires were completed not individually but in class groups. The research design was based on voluntary consent. It can be assumed that some adolescents with psychological problems might have been not truthful enough to answer the questions and/or could have avoided the survey explicitly or implicitly. This may be a consequence of the social desirability, consent, and memory recall biases that characterize many psychological studies involving voluntary, self-administered questionnaires [65,66,67,68]. Secondly, the study was not a longitudinal one, which makes it impossible to assess the stability of symptoms and content structure with age. Thirdly, one serious limitation of our study is the lack of complete information about the availability of Internet use for the respondents. However, according to our general observations, almost all urban adolescents in Russia have smartphones, and broadband Internet access is very cheap and available even to the poorest segments of the population. On the other hand, some parents may limit adolescents’ access to games and social networks (parental control function). The exact proportion of such adolescents was not taken into account in this study. Finally, we did not assess the type of user devices (desktop computers/smartphones/tablets), technologies (e.g., a possible crossover between PUgame and PUsocial in games using active social communication), specific games, and social networks.

## 5. Conclusions

The prevalence of generalized PIU among adolescents in Central Siberia, as assessed by the CIAS questionnaire, produced a figure of 7.2%; the prevalence of PUgame assessed by the GASA questionnaire was 10.4%; the prevalence of PUsocial assessed by the SMDS questionnaire was 8.0%. Several significant gender differences in specific types of PIU were identified: the predominance of gaming addiction among the boys and social networking addiction among the girls. According to the above indicators, urban adolescents in Central Siberia did not differ significantly from their peers from Asian and European countries. However, unlike the situation in most other countries, the prevalence of generalized PIU was higher among Russian girls compared to the boys.

Whereas the boys aged 12–18 showed no significant changes in the overall prevalence of PIU and content consumption patterns, the girls showed an increase in the relative prevalence of undifferentiated content consumption unrelated to computer games and social media addiction, along with an increasing prevalence of PIUgen with age. 

The results of the structural equation modelling, as well as the data of logistic regression and correlation analysis, suggest the presence of two possible patterns of psychosocial problems in PIU: the first is characteristic of both PIUgen and PUsocial, and the second, which is significantly different, is characteristic of PUgame. In terms of psychological construct, PUgame and PUsocial appear to be distinct nosological entities. PUgame should be regarded as a subtype of general gaming addiction, possibly, with some features of problem gambling. At the same time, social networking addiction may show signs of a “fear of missing out” disorder and may also be a cause or a consequence of more general anxiety and depression disorders.

Given the main findings of the present study, the identified different types of addictive online behavior had a low degree of intercorrelation in terms of age-gender and psychological characteristics. This fact suggests that the concept of generalized Internet addiction (undifferentiated by content) has insignificant justification, while the term “Internet addiction” can be misused and misinterpreted in this regard [19,20]. Additional research is needed for better identification of the commonalities and differences in different types of addictive online behavior in the context of consumed content, used devices and technologies (desktop computers, tablets, and smartphones), and changes in addiction over time (the stability of symptoms and the possibility of remission and relapse). It is extremely important to carry out future research with the use of representative sampling and longitudinal design.

## Figures and Tables

**Table 1:** Psychometric characteristics of the questionnaires included in the study.

Questionnaire	Cronbach’s Alpha	χ^2^ df, *p*	CFI	TLI	RMSEA (90% CI)
Chen Internet Addiction Scale (CIAS)	0.909	5890 299 ***	0.837	0.823	0.064 (0.063–0.066)
Game Addiction Scale for Adolescents (GASA)	0.917	688 14 ***	0.968	0.952	0.103 (0.097–0.110)
Social Media Disorder Scale (SMDS)	0.679	464 27 ***	0.898	0.865	0.060 (0.055–0.065)
Strengths and Difficulties Questionnaire (SDQ)	0.656	8272 275 ***	0.528	0.485	0.080 (0.079–0.081)

Note: CFI—comparative fit index; TLI—Tucker–Lewis index; RMSEA—root mean square error of approximation; CI—confidence interval. ***: *p* < 0.001.

**Table 2:** Descriptive statistics for major study variables.

Variables	All Participants	Boys	Girls	*p* (Boys vs. Girls)
Age 12–14	2217	1003 (45.2%)	1214 (54.8%)	-
Age 15–18	2297	1089 (47.4)	1208 (52.6%)	-
Total	4514	2092 (46.4%)	2422 (53.6%)	-
City				
Krasnoyarsk	2901	1327 (45.7%)	1574 (54.3%)	-
Abakan	1400	674 (48.1%)	726 (51.9%)	-
Kyzyl	213	91 (42.7%)	122 (57.3%)	-
Ethnicity				
Russians	3546	1656 (46.7%)	1890 (53.3%)	-
Khakass	164	66 (40.2%)	98 (59.8%)	-
Tuvans	397	179 (45.1%)	218 (54.9%)	-
Others	407	191 (46.9%)	216 (53.1%)	-
CIAS results (*n* = 4514)				
Chen Internet addiction Scale (CIAS)	44.3 ± 12.5	42.5 ± 12.0	45.8 ± 12.7	0.009
Generalized problematic Internet use (PIUgen), CIAS ≥ 65	324 (7.2%)	109 (5.2%)	215 (8.9%)	<0.001
GASA results (*n* = 4514)				
Game Addiction Scale for Adolescents (GASA)	9.6 ± 6.7	12.1 ± 6.03	7.5 ± 6.3	<0.001
Problematic computer game use (PUgame), 4/7 GASA items endorsed	471 (10.4%)	329 (15.7%)	142 (5.9%)	<0.001
SMDS results (*n* = 4514)				
Social Media Disorder Scale (SMDS)	1.7 ± 1.8	1.1 ± 1.5	2.1 ± 1.9	<0.001
Problematic social media use (PUsocial) 5/9 SMDS items endorsed	359 (8.0%)	73 (3.5%)	286 (11.8%)	<0.001
Strengths and Difficulties Questionnaire (SDQ) results (*n* = 4514)				
Total difficulties	11.4 ± 5.4	10.4 ± 5.2	12.3 ± 5.4	<0.001
Emotional symptoms	3.1 ± 2.5	2.1 ± 2.1	3.8 ± 2.5	<0.001
Hyperactivity	3.3 ± 2.0	3.1 ± 2.0	3.4 ± 2.1	<0.001
Conduct problems	2.3 ± 1.5	2.3 ± 1.5	2.3 ± 1.5	0.955
Peer problems	2.8 ± 1.8	2.8 ± 1.8	2.8 ± 1.8	0.687
Prosocial behavior	7.1 ± 2.1	6.8 ± 2.2	7.4 ± 2.1	<0.001

Note: data are presented as *n* (%) and mean ± SD. Pearson χ^2^ test and Student’s *t*-tests were used, respectively.

**Table 3:** Age-specific features of generalized and specific PIU with stratification by gender (*n* = 4514).

	Age 12–14				Age 15–18			
	All *n* = 2217	Boys *n* = 1003	Girls *n* = 1214	*p* (Boys vs. Girls)	All *n* = 2297	Boys *n* = 1089	Girls *n* = 1208	*p* (Boys vs. Girls)
Generalized problematic Internet use (PIUgen), CIAS ≥ 65	150 (6.8%)	57 (5.7%)	93 (7.7%)	0.078 χ^2^ = 3.1	174 (7.6%)	52 (4.8%)	122 (10.1%)	<0.001 χ^2^ = 22.4
Game users (computer game playing during past month)	1795 (81.0%)	930 (92.7%)	865 (71.3%)	<0.001 χ^2^ = 162.9	1809 (78.8%)	1089 (89.5%)	720 (59.6%)	<0.001 χ^2^ = 263.7
Problematic computer game use (PUgame), 4/7 GASA items endorsed	261 (11.8%)	174 (17.4%)	87 (7.2%)	<0.001 χ^2^ = 53.8	210 (9.1%)	155 (14.2%)	55 (4.6%)	<0.001 χ^2^ = 63.5
Computer game addiction, 7/7 GASA items endorsed	21 (1.0%)	11 (1.1%)	10 (0.8%)	0.660 χ^2^ = 0.2	27 (1.2%)	21 (1.9%)	6 (0.5%)	0.003 χ^2^ = 8.9
Social media users (social media use during past year)	2112 (95.3%)	933 (93.0%)	1179 (97.1%)	<0.001 χ^2^ = 19.5	2224 (96.8%)	1045 (96.0%)	1179 (97.6%)	0.034 χ^2^ = 4.5
Problematic social media use (PUsocial), 5/9 SMDS items endorsed	214 (9.7%)	44 (4.4%)	170 (14.0%)	<0.001 χ^2^ = 57.1	145 (6.3%)	29 (2.7%)	116 (9.6%)	<0.001 χ^2^ = 45.5
Social media addiction, 9/9 SMDS items endorsed	10 (0.5%)	4 (0.4%)	6 (0.5%)	0.988 χ^2^ < 0.1	8 (0.4%)	3 (0.3%)	5 (0.4%)	0.836 χ^2^ < 0.1

**Table 4:** Contingency table showing the overlap between generalized problematic Internet use (generalized PIU) and problematic computer game use (PUgame), *n* = 4514.

Generalized Problematic Internet Use (Generalized PIU)	Problematic Computer Game Use (PUgame)		
	No	Yes	Total
No	3839 (85.1%)	351 (7.8%)	4190 (92.8%)
Yes	204 (4.5%)	120 (2.7%)	324 (7.2%)
Total	4043 (89.6%)	471 (10.4%)	4514 (100%)

**Table 5:** Contingency table showing the overlap between generalized problematic Internet use (generalized PIU) and problematic social media use (PUsocial), *n* = 4514.

Generalized Problematic Internet Use (Generalized PIU)	Problematic Social Media Use (PUsocial)		
	No	Yes	Total
No	3980 (88.2%)	210 (4.6%)	4190 (92.8%)
Yes	175 (3.9%)	149 (3.3%)	324 (7.2%)
Total	4155 (92.1%)	359 (7.9%)	4514 (100%)

**Table 6:** Contingency table showing overlap between problematic computer game use (PUgame) and problematic social media use (PUsocial), *n* = 4514.

Problematic Computer Game Use (PUgame)	Problematic Social Media Use (PUsocial)		
	No	Yes	Total
No	3792 (84.0%)	251 (5.6%)	4043 (89.6%)
Yes	363 (8.0%)	108 (2.4%)	471 (10.4%)
Total	4155 (92.0%)	359 (8.0%)	4514 (100%)

**Table 7:** Logistic regression estimates for generalized problematic Internet use (CIAS scale), problematic computer game use (GASA scale), and problematic social media use (SMDS scale), according to psychosocial problems evaluated with SDQ in Russian adolescents (crude and adjusted odds ratios, *n* = 4514).

	Generalized Problematic Internet Use (PIU)			Problematic Computer Game Use (PUgame)			Problematic Social Media Use (PUsocial)		
	OR Crude	OR Adjusted	*p*	OR Crude	OR Adjusted	*p*	OR Crude	OR Adjusted	*p*
Total difficulties	4.58 3.62–5.79	4.47 3.52–5.67	<0.001	3.02 2.45–3.71	3.79 3.05–4.71	<0.001	3.84 3.06–4.81	3.48 2.76–4.38	<0.001
Emotional symptoms	3.83 3.05–4.82	3.65 2.89–4.64	<0.001	1.77 1.45–2.16	2.89 2.32–3.60	<0.001	4.07 3.26–5.07	3.31 2.63–4.16	<0.001
Hyperactivity	4.50 3.36–6.03	4.56 3.38–6.13	<0.001	3.04 2.30–4.00	3.55 2.66–4.75	<0.001	4.09 3.07–5.46	3.90 2.89–5.24	<0.001
Conduct problems	2.95 2.32–373	3.10 2.44–3.94	<0.001	2.34 1.91–2.88	2.34 1.90–2.90	<0.001	2.38 1.89–3.00	2.44 1.93–3.09	<0.001
Peer problems	2.12 1.73–2.73	2.17 1.73–2.73	<0.001	1.25 1.18–1.31	1.25 1.19–1.32	<0.001	1.14 1.08–1.21	1.14 1.08–1.21	<0.001
Low level of prosocial behavior	1.83 1.36–2.46	1.97 1.46–2.67	<0.001	1.86 1.44–2.40	1.71 1.32–2.22	<0.001	1.13 0.81–1.56	1.33 0.95–1.85	0.476 (OR crude) 0.098 (OR adjusted)

Note: ORs with 95% confident intervals are shown. Crude ORs were adjusted for sex, age, city (Krasnoyarsk, Abakan, Kyzyl), and ethnicity (Russians, Khakass, Tuvans, others).

**Table 8:** Correlation matrix of studied variables.

Variables	CIAS	GASA	SMDS	Total Difficulties	Emotional Symptoms	Hyperactivity	Conduct Problems	Peer Problems	Prosocial Behavior
CIAS	-								
GASA	0.292	-							
SMDS	0.592	0.159	-						
Total difficulties	0.430	0.181	0.367	-					
Emotional symptoms	0.376	0.043	0.370	0.767	-				
Hyperactivity	0.358	0.176	0.271	0.698	0.346	-			
Conduct problems	0.234	0.161	0.194	0.597	0.270	0.364	-		
Peer problems	0.156	0.163	0.099	0.588	0.309	0.180	0.211	-	
Prosocial behavior	0.088	0.134	0.023	0.174	0.007	0.147	0.188	0.187	-

Note: All values are significant at *p* < 0.001, except for underlined values (*p* > 0.05). Gray color marks Spearman’s rank correlation coefficient > 0.3.

**Table 9:** Regression weights of multiple regression model for generalized problematic Internet use (PIUgen), *n* = 4514.

Variables	Estimate	Approximate Standard Error	Critical Ratio	*p*
Female	2.054	0.363	5.664	<0.001
Age	0.640	0.346	1.850	0.064
CIAS				
Total difficulties	2.847	0.666	4.278	<0.001
Emotional symptoms	4.701	0.486	9.669	<0.001
Hyperactivity	5.629	0.743	7.580	<0.001
Conduct problems	2.539	0.491	5.170	<0.001
Peer problems	0.814	0.414	1.964	0.049
Prosocial behavior	2.550	0.555	4.592	<0.001

**Table 10:** Regression weights of multiple regression model for problematic video game use (PUgame), *n* = 4514.

Variables	Estimate	Approximate Standard Error	Critical Ratio	*p*
Female	−5.023	0.190	−26.433	<0.001
Age	−1.128	0.181	−6.223	<0.001
CIAS				
Total difficulties	1.907	0.349	5.469	<0.001
Emotional symptoms	0.897	0.255	3.521	<0.001
Hyperactivity	1.403	0.389	3.605	<0.001
Conduct problems	0.711	0.257	2.764	0.006
Peer problems	0.697	0.217	3.211	0.001
Prosocial behavior	0.885	0.291	3.041	0.002

**Table 11:** Regression weights of multiple regression model for problematic social media use (PUsocial), *n* = 4514.

Variables	Estimate	Approximate Standard Error	Critical Ratio	*p*
Female	0.785	0.051	15.502	<0.001
Age	−0.153	0.048	−3.163	0.002
CIAS				
Total difficulties	0.201	0.093	2.166	0.030
Emotional symptoms	0.709	0.068	10.442	<0.001
Hyperactivity	0.664	0.104	6.406	<0.001
Conduct problems	0.343	0.069	5.011	<0.001
Peer problems	−0.010	0.058	−0.178	0.859
Prosocial behavior	0.192	0.078	2.479	0.013

## Data Availability

The datasets generated for this study are available on request to the corresponding author.

## Abbreviations

PIU	problematic Internet use
PIUgen	generalized problematic Internet use
PUgame	problematic video game use
PUsocial	problematic social media use
CIAS	Chen Internet Addiction Scale
GASA	Game Addiction Scale for Adolescents
SMDS	Social Media Disorder Scale
SDQ	Strengths and Difficulties Questionnaire
RMSEA	root mean square error of approximation
CFI	comparative fit index
TLI	Tucker–Lewis index
OR	odds ratio
